# An Energy Landscape for Elemental Carbon

**DOI:** 10.1021/acs.jpclett.6c02357

**Published:** 2026-08-21

**Authors:** Vilmos Neuman, Soon Woo Park, Mark Wilson, David J. Wales

**Affiliations:** † Department of Chemistry, Physical and Theoretical Chemistry Laboratory, 6396 University of Oxford, South Parks Road, Oxford OX1 3QZ, U.K.; ‡ Yusuf Hamied Department of Chemistry, 2152 University of Cambridge, Lensfield Road, Cambridge CB2 1EW, U.K.

## Abstract

The potential energy surface of an 8-atom periodic cell of carbon is explored using discrete path sampling with the energy, forces, and stress tensor represented by the MACE-MATPES-r2SCAN-0 potential evaluated via an interface to the Atomic Simulation Environment. The resulting enthalpy landscape is explored as a function of pressure and compared to the experimental phase diagram. Several interesting pathways between different phases are analyzed in detail, including symmetry analysis for degenerate rearrangements of periodic crystals. We also considered the effect of a dispersion correction on the energetics and structure of selected crystals. The approach employed here is generally applicable for materials and condensed phase modeling.

## Graphical abstract

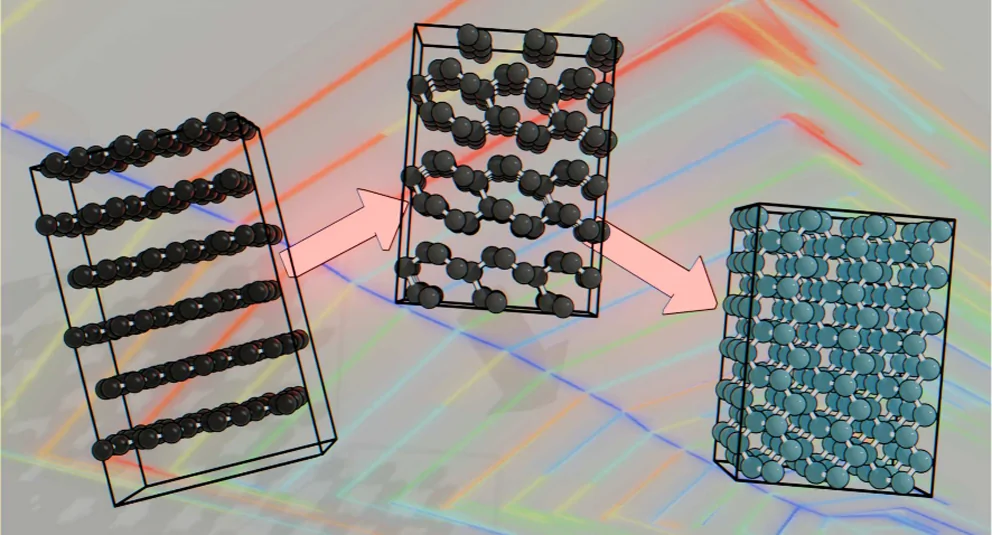

The potential energy surface (PES), which governs emergent properties such as structure, dynamics, and thermodynamics, is a central theme in chemical physics.^1,2^ Coarse grained as a database of minima interconnected by first-order saddle points (transition states),^3^ the PES provides a natural description of the pathways between distinct metastable (local minimum) structures. Such multiple metastable structures, where interconversions correspond to phase transitions, are ubiquitous in condensed matter polymorphism. In particular, elemental carbon not only supports various crystalline allotropes, such as diamond and graphite, but also possesses rich polyamorphism and extensive cluster chemistry.^4–11^

Exploring the energy landscape of a solid state system requires periodic boundary conditions, which introduce additional degrees of freedom associated with the size and shape of the periodic unit cell.^12–16^ For a pathway between periodic graphite and diamond, for example, the cell must change significantly, since the two allotropes have markedly different number densities. The algorithms used to search the landscape through geometry optimization must therefore include the cell degrees of freedom, as in previous work.^14,17–20^ In this contribution, we employ an interface for landscape exploration using the OPTIM^21^ and PATHSAMPLE^22^ programs, where the potential energy, forces, and stress tensor are obtained from the Atomic Simulation Environment (ASE).^23^ This interface provides access to a wide array of recently developed data-driven potentials, which have become promising tools for modeling condensed phase and materials phenomena.^24^ A detailed description and validation of the ASE interface employed here will be presented elsewhere.^25^ Using this implementation, we survey the energy landscape of an 8-atom periodic carbon cell described by the MACE-MATPES-r2SCAN-0 foundation model.^24,26^ In future work we will extend this investigation to a wider array of systems and system sizes; the present results illustrate the details that become accessible when such models are combined with geometry optimization tools for exploring the global landscape.

Since the selected foundation model was fine-tuned on r2SCAN^27^ data, its ability to capture long-range London dispersion interactions is limited. To quantify and partly remedy this limitation we adopt a two-body truncation of the D3­(BJ)^28^ correction with coefficients^29^ optimized for the r2SCAN functional. This recipe amounts to adding an empirical term of the form

1 $$E_{\mathrm{disp}}^{D3}=-\underset{\left\langle ij\right\rangle }{\sum }\underset{n=6,8}{\sum }s_{n}\frac{C_{n}^{ij}}{R_{ij}^{n}}f_{\mathrm{damp}}^{\left(n\right)}\left(R_{ij}\right)$$

where the first sum runs over all atom pairs *ij*, *s*_*n*_ is a fitted scaling parameter, $C_{n}^{ij}$ is the tabulated geometry dependent dispersion coefficient for the elements of atoms *i* and *j*, *R*_*ij*_ is the distance between atoms *i* and *j*, and $f_{\mathrm{damp}}^{\left(n\right)}\left(R_{ij}\right)$ is the Becke-Johnson (BJ) damping function.^30^ This is an additive dispersion correction, performed in analogy to dispersion-corrected DFT methods, in which the underlying potential describing the short-ranged interactions is left unchanged and only the parameters of the additive term are matched to the functional on which the force field was trained. This approach follows analogous treatments in the machine learning potential literature for fitting potentials.^24,31^ Since OPTIM is interfaced through ASE, such additive dispersion corrections can be applied straightforwardly to machine learning potentials within this architecture. Figure 1a,b shows the potential energy disconnectivity graphs^32,33^ for the core and dispersion corrected potentials, respectively. In these graphs, energy increases vertically, and two minima are joined into a “superbasin” when a pathway interconverts them below the threshold energy level corresponding to the vertical height of the branching point. Both graphs are relatively funnelled, with the central funnel containing the global minimum being relatively kinetically isolated and corresponding to graphitic structures. For the core potential, this central funnel features numerous graphitic minima, all related by polytypism (i.e., different stacking patterns) and/or somewhat different interlayer spacings. These minima are all separated by small barriers corresponding to reorganization of the stacking. Adding the dispersion correction appears to reduce the number of graphitic structures in the central funnel, strongly favoring a single interlayer spacing and stacking structure. We note that the dispersion correction reduces the interlayer spacing of graphite with *P*6_3_/*mmc* symmetry in Hermann-Mauguin notation from 3.69 Å to 3.48 Å. However, this spacing is still above the crystallographic value of 3.35 Å. A similar effect, namely multiple stable graphitic structures with incorrect layer spacings, has previously been reported for other data driven models.^100^

**Figure 1:**
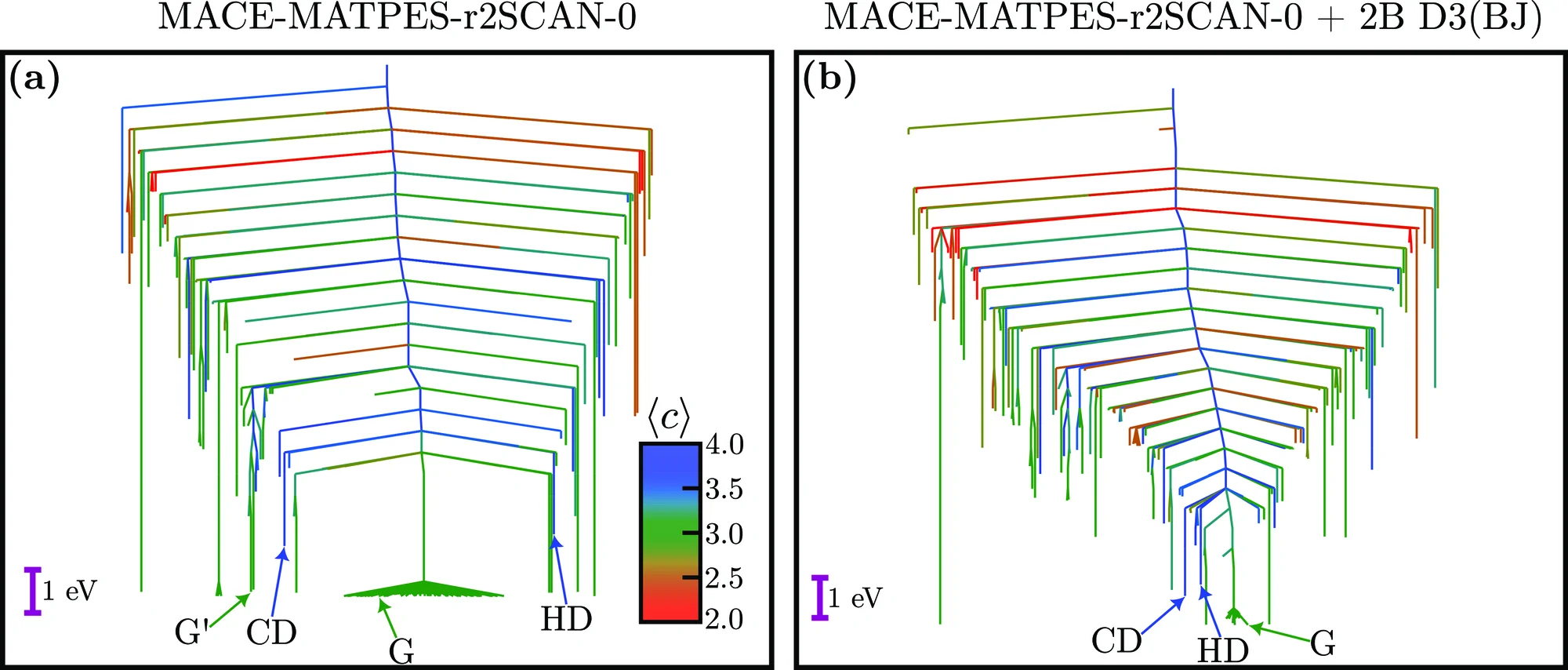
Potential energy disconnectivity graphs for the periodic 8-atom carbon cell described by the MACE-MATPES-r2SCAN-0 foundation model alone (panel a) and with the two-body D3­(BJ) dispersion correction added (panel b). Minima are colored according to the mean coordination number of the structure, ⟨*c*⟩, and those labeled CD, HD, and G correspond to cubic diamond, hexagonal diamond, and graphite, respectively and G′ of panel a labels the diagonally stacked graphite configuration of Figure 2. These results are for zero pressure with a variable cell. Bonds are defined using a 1.7 Å distance cutoff.

Both graphs feature kinetic traps with three-coordinate carbon atoms, which consist of local minima that are separated by large barriers from the main funnel, corresonding to graphitic structures. These traps exist for two reasons, both of which make the apparent landscape artificially frustrated. The first effect is likely simply a reflection of finite sampling, where local minima have been identified as connected to the kinetic transition network, but lie behind large barriers, because lower-energy pathways linking them into the network have not yet been found. As discrete path sampling proceeds, more pathways are identified, and we therefore often find that a better path is located for a particular pair of local minima, reducing the barrier displayed in the disconnectivity graph. The second reason is genuinely informative about the nature of the energy landscape under periodic boundary conditions. Traps can arise when the graphitic planes are oriented at an angle to the basal plane of the cell, and hence to the orientation of the layers in the central funnel, as shown in Figure 2. A similar effect has previously been reported for the FCC crystal in the crystallization landscape of a Lennard-Jones fluid.^34^ Note that these misoriented graphite structures must also differ from those in the central funnel in either interlayer spacing or stacking pattern, otherwise they would lie in the central funnel, since we lump together equivalent permutation-inversion isomers (see Computational Methods). These two minima cannot interconvert via a simple rotation, which one might expect would possess a relatively low barrier, since this process would break intralayer bonding at the cell boundary due to the nature of periodic boundary conditions. An effective “selection rule” may be developed to describe this restriction, which is presented in the Supporting Information, together with an illustration of the lowest energy pathway connecting the horizontal and diagonal forms.

**Figure 2:**
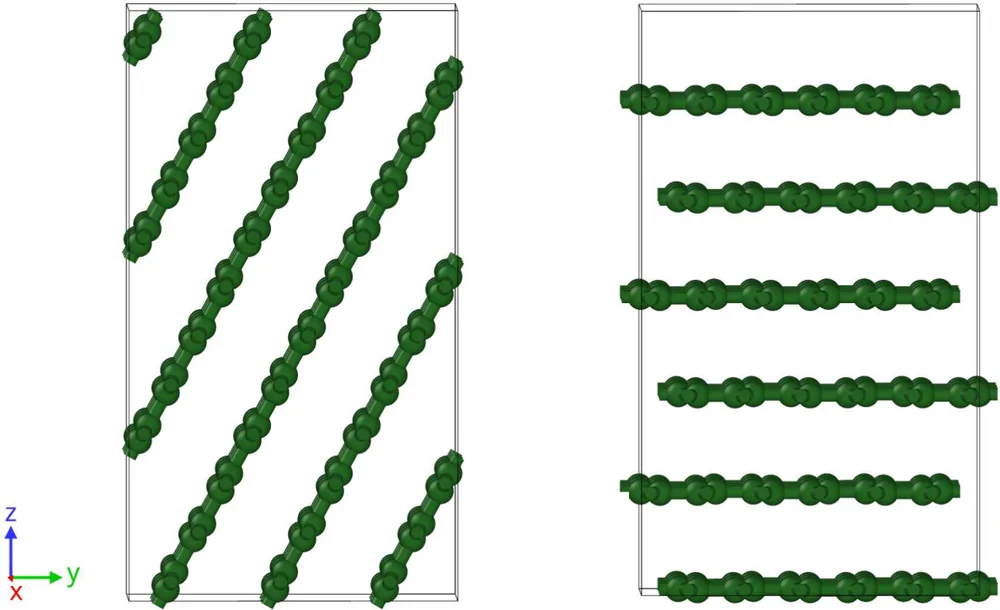
Ball and stick models showing two distinct graphitic minima in which the stacked layers are oriented at an angle with respect to each other and are separated by high barriers. 3 × 3 × 3 supercells are shown for clarity.

Both landscapes feature interesting minima and interconversion pathways, and we describe some of them below. Figure 3a shows a direct graphite-to-cubic-diamond pathway in which the graphitic layers approach and shift relative to one another, then buckle into the chair conformation, forming connected chair bilayers at the transition state. The bilayers then approach each other and contract to form the Fd3̅m symmetry cubic diamond. The barrier is 0.671 eV/atom, somewhat higher than comparable values reported elsewhere,^20,40^ suggesting either that a lower energy pathway exists or a limitation of the potential. We note that this mechanism is certainly not the most favorable pathway for the phase transition of the bulk, which is much more likely to occur via nucleation and growth. However, the structural motif identified here might act as a possible nucleus in a stepwise mechanism. Future work will focus on exploring whether this rearrangement can be realized as a critical step in diamond nucleation.

**Figure 3:**
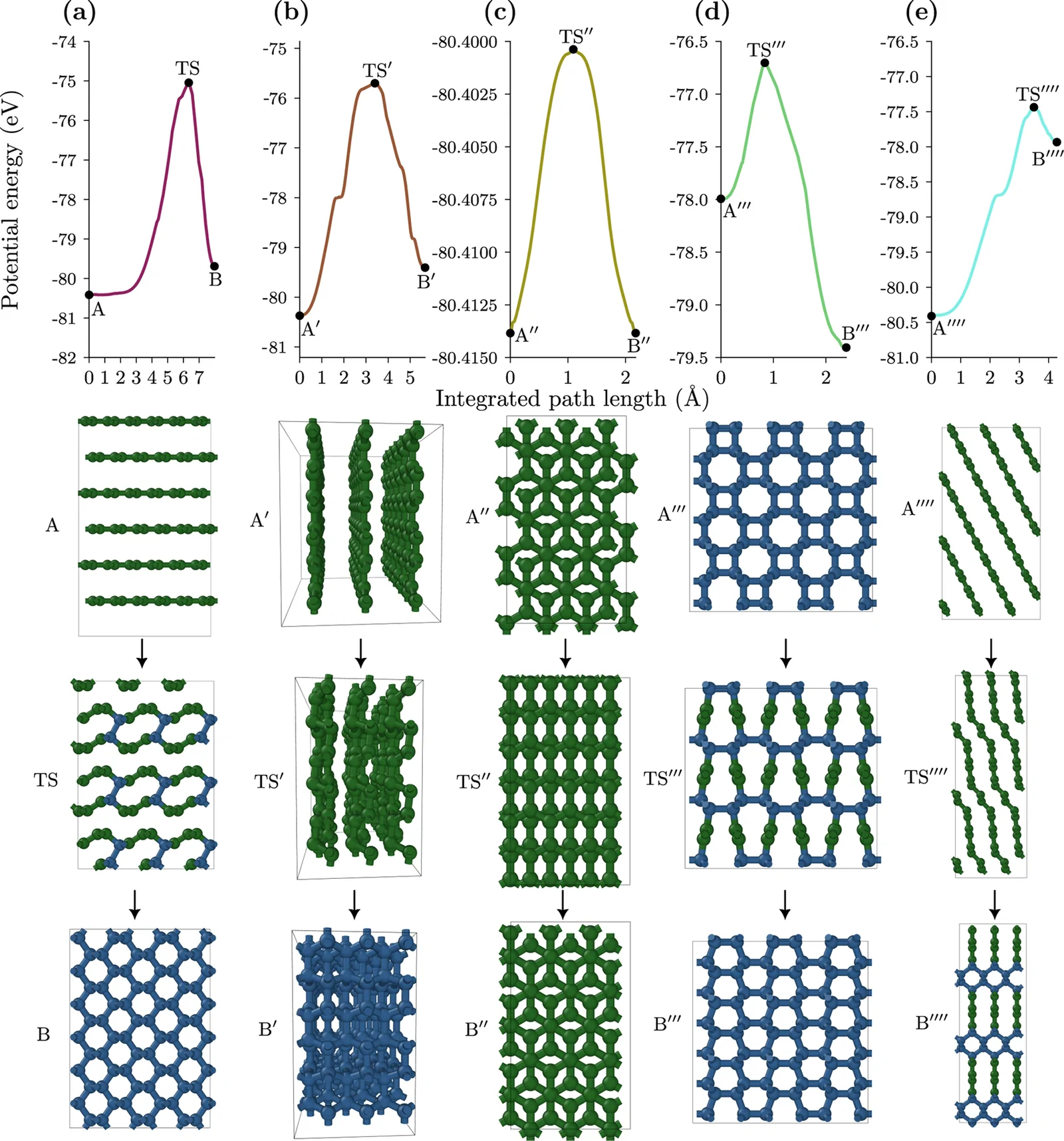
Five selected pathways (a)–(e) from the potential energy landscape described by the MACE-MATPES-r2SCAN-0 foundation model with D3 dispersion correction. 3 × 3 × 3 supercells are shown for clarity and three- and four-coordinate atoms are shown in green and blue, respectively.

The path shown in Figure 3b terminates in hexagonal diamond (lonsdaleite) and is a useful example for highlighting the structural relationship between the hexagonal and cubic forms. It begins from an AA-stacked graphite, which may form simply from the most prevalent Bernal-stacked (AB) polytype by sliding. Along this pathway, the honeycomb layers buckle into the “boat” conformation at the transition state. The layers then approach one another, accompanied by a reduction in cell volume, to yield a structure that may be thought of as layers of carbon puckered into the chair conformation, related by a mirror plane, with the interlayer bonding forming six-membered rings in the “boat” conformation. The existence of this mirror plane is crucial and stems from the graphitic layers not sliding over one another along the pathway, in contrast to the previous mechanism in Figure 3a. As a result, lonsdaleite is an AB-stacked system (see the B′ structure in Figure 3b), whereas cubic diamond is ABC-stacked.

The third pathway in Figure 3c is a degenerate rearrangement,^35^ which provides an example of how symmetry analysis for molecular systems can be extended to solids.^36^ By definition, the transition state is a first-order saddle; therefore, it possesses a single imaginary frequency Hessian eigenmode. Stepping along this mode in either direction away from the transition state leads the system onto the two steepest-descent paths, which connect two minima. Since there are only two such paths,^1,37^ any symmetry operation of the transition state must either be a symmetry operation of the steepest-descent paths, including the minima where they terminate, leaving them unchanged, or it must exchange them. Hence, a transition state can possess a symmetry element that the minima lack only if that operation interconverts them, in which case the pathway corresponds to a degenerate rearrangement.^35^ Here, the *Fmmm* symmetry transition state connects two equivalent representations of *P*6_3_/*mmc* graphite that interconvert by sliding over one another by half of a bond length. The transition state therefore possesses additional centering symmetries, such as the (1/2, 0, 1/2) translation, which interconvert the two minima, and indeed any pair of corresponding structures along the steepest-descent paths. This result is reflected in the symmetric energy profile in Figure 3c. We note that such symmetry rules are well-known for molecules and hence for point group operations.^37,38^ The situation here is different since the transition state possesses no additional point group symmetry, but rather additional translational symmetry associated with the Bravais lattice, so this result is distinct from the isolated molecule case and shows how these symmetry considerations extend to space groups.

The pathway shown in Figure 3d, involves the theoretically predicted body-centered tetragonal carbon, which consists of four-coordinate carbons bonded into C_4_ square rings, yielding a mixture of corner- and edge-sharing tetrahedra. The structure has *I*4/*mmm* symmetry and is predicted to form from carbon nanotubes under pressure.^39^ Umemoto et al.^39^ proposed that it may serve as an intermediate in the conversion of graphite to hexagonal diamond, to which it is very closely related. The pathway, which yields the lonsdaleite structure, highlights this relationship as it amounts to a rearrangement of half of the atoms through a transition state where these atoms are three-coordinate, resulting in a rearrangement corresponding to a change in the buckling pattern.

The final pathway highlighted in Figure 3e is for an AB stacked graphite containing a stacking defect, in which every alternate layer is shifted by half a C–C bond length. As a result, when viewed along the “soft” axis, two atoms from the layer below appear inside each upper hexagon of the honeycomb. As the unit cell volume decreases, the layers approach one another and buckle, forming a kinked transition state. This transition state contains segments containing two hexagons oriented nearly perpendicular to the basal plane, as well as a second set of regions with two hexagons tilted at approximately 60° to the first one. On further compression, the tilted layers approach one another, while the other layers become fully orthogonal to the basal plane, giving rise to alternating layers of AA stacked graphite and cubic diamond joined through an interface. The resulting structure is essentially a crystalline diamond-graphite composite or “diaphite”.^8,9,40–42^ This is an interesting potential allotrope, featuring an unusually short (2.62 Å) interlayer spacing here within the graphitic domain, which is directly governed by the structure of the diamond domains and the interface (often termed “gradia”). Diaphites have been proposed as materials with potential useful technological applications,^40^ so future work could model the formation of these structures in larger systems that would be more representative of the bulk.

We analyze the effect of pressure in the enthalpy landscapes by adding to the Cauchy stress tensor, which is given by

2 $$\Sigma^{\mathrm{Cauchy}}=\left(\begin{array}{lll} \Sigma_{x} & \tau_{xy} & \tau_{xz}\\ \tau_{xy} & \Sigma_{y} & \tau_{yz}\\ \tau_{xz} & \tau_{yz} & \Sigma_{z} \end{array}\right)$$

where Σ_*x*_, Σ_*y*_, and Σ_*z*_ are the normal stresses and τ_*xy*_, τ_*xz*_, and τ_*yz*_ are the shear stresses. An isotropic pressure *p* is then incorporated by adding it along the diagonal

3 $$\Sigma^{\mathrm{total}}=\Sigma^{\mathrm{Cauchy}}+pI$$

where ***I*** is the second-order identity tensor. Extracting the cell derivatives from the modified stress and performing the geometry optimizations with these derivatives yields the enthalpy landscape defined by *H* = *E* + *pV*. As for the potential energy surface, this enthalpy landscape does not depend on temperature, masses, or coordinate system. The implementation is straightforward^17^ and the OPTIM program allows enthalpy surfaces to be surveyed at both positive and negative pressures. Figure 4a,b shows the enthalpy landscapes of the MACE foundation model at 10 GPa and −1 GPa, respectively. The landscape appears more sensitive to negative pressures, which motivated the choice of these two values. Panel (a) shows that cubic diamond becomes the global minimum at 10 GPa, consistent with the phase diagram at low temperature and suggesting that the enthalpy likely dominates the free energy in this temperature regime. At a negative pressure of −1 GPa, the disconnectivity graph in Figure 4b shows that the graphitic funnels become more stable relative to diamond when compared to the zero pressure case in Figure 1a, again consistent with the expected phase behavior. The potential energy or enthalpy differences between the most stable graphitic and cubic diamond structures in each of the landscapes are summarized in Table 1. Interestingly, upon addition of the D3 correction, the energy of cubic diamond decreases more than for graphite, likely because of its higher density. This result is consistent with previous reports showing that dispersion is also relevant in the diamond structure.^43^ Importantly, although the energy of the diamond structure is lowered significantly, the structure itself is effectively unchanged, as the C–C bond length only contracts from 1.55 Å to the crystallographic value of 1.54 Å upon addition of the dispersion correction. As discussed above, the correction affects the interlayer spacing of graphite far more strongly. We therefore conclude that dispersion is relevant in both crystals, but is structure determining only in graphite. Adding pressure has structural effects as well as energetic reordering of the minima. For example, at −1 GPa the global minimum graphitic structure is ABC-stacked, with an extended interlayer spacing of 3.89 Å. In contrast, at 10 GPa, the interlayer spacing is only 3.26 Å.

**Figure 4:**
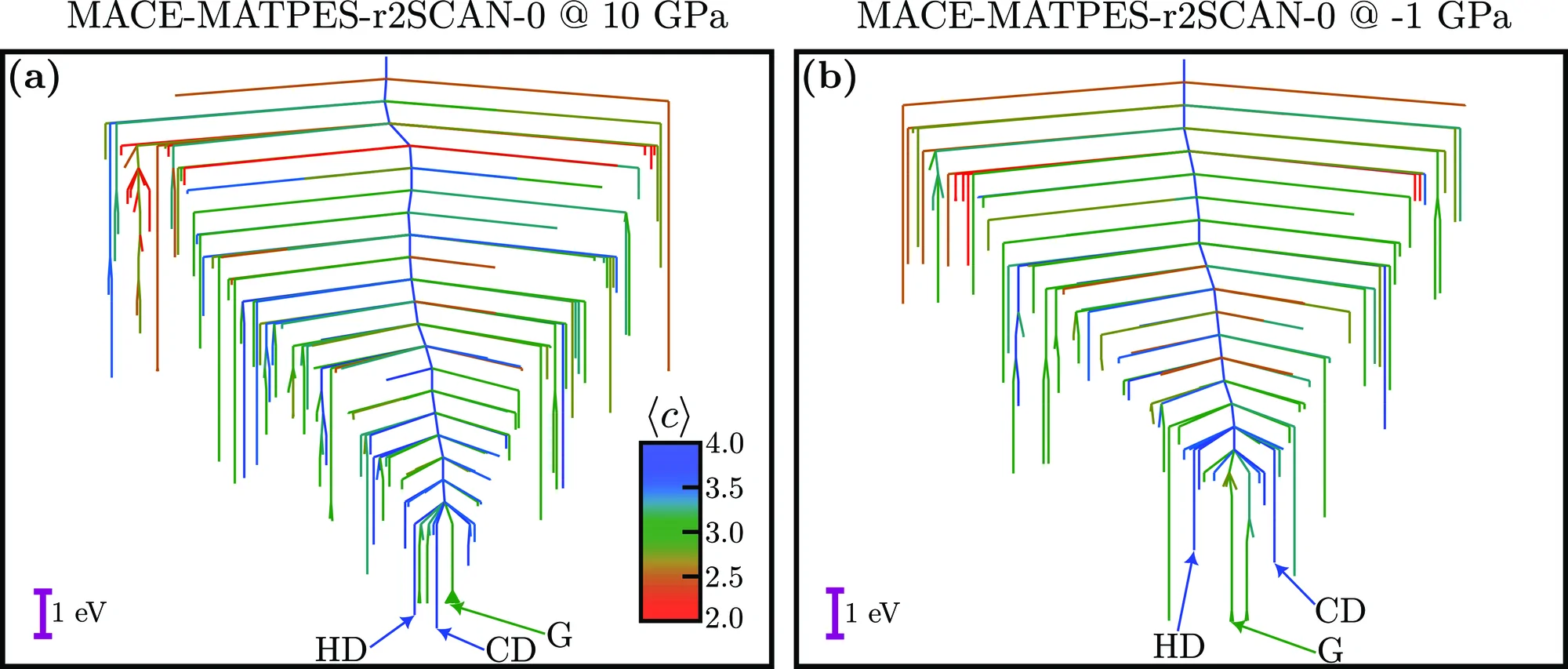
Enthalpy disconnectivity graphs for the periodic 8-atom carbon cell described by the MACE-MATPES-r2SCAN-0 foundation model at 10 GPa (panel a) and −1 GPa (panel b). Minima are colored according to the mean coordination number of the structure, ⟨*c*⟩, and those labeled CD, HD, and G correspond to cubic diamond, hexagonal diamond, and graphite, respectively. Bonds are defined using a 1.7 Å distance cutoff.

**Table 1:** Enthalpy Difference Δ*H* = *H*_diamond_ – *H*_graphite_ between Cubic Diamond and Graphite^a^

	MACE	MACE+D3	MACE@10 GPa	MACE@–1 GPa
Δ*H* (eV/atom)	0.135	0.091	–0.149	0.161

a At zero pressure Δ*H* reduces to the potential energy difference Δ*E*; the two finite-pressure columns include the *pV* term.

In this Letter, we have used OPTIM^21^ and PATHSAMPLE^22^ with an ASE^23^ interface for construction of kinetic transition networks and explored the energy landscape of a small cell of elemental carbon. The energy landscapes are compared for a dispersion correction to a MACE foundation model^24^ and at different pressures. The effect of pressure was found to be in good agreement with the phase diagram, and the dispersion correction also led to meaningful results, such as the reduction in the interlayer spacing of graphite. Several pathways have been highlighted, including a concerted graphite-to-diamond phase transition, which might provide clues to the formation of a critical nucleus in the phase transition of the bulk. We also presented a symmetry result for degenerate rearrangements involving space group symmetries, which extends previous results from point group symmetry. To build on this detailed analysis of a small supercell, future work will consider larger system sizes to predict realistic nucleated phase transition mechanisms. We also plan to explore negative pressure phenomena, such as the rupturing of silica glass.^44^

## Computational Methods

All the energy landscapes were explored using geometry optimization techniques in the OPTIM program,^21^ with parallel explorations organized by the PATHSAMPLE^22^ program. Databases of stationary points were expanded using the discrete path sampling approach,^45^ in which maxima from initial doubly-nudged^46,47^ elastic band^48,49^ paths are taken as transition state candidates and refined to true transition states by gradient-only hybrid eigenvector-following.^50–53^ The connected minima are assigned via approximate steepest-descent paths, and complete multistep paths are obtained using iterative searches guided by the missing connection algorithm.^54^ This procedure produces a kinetic transition network^55–58^ of minima interconnected by first order saddles.

Periodic boundary conditions and variable cells introduce additional degrees of freedom,^12–16^ which we treated in terms of the unit cell lengths and angles, as described in previous work.^17^ In particular, the doubly-nudged elastic band approach that is used to propose candidates for refinement, is closely related to the solid-state nudged elastic band procedure of Henkelman and co-workers.^14,18–20^ The initial database for the core MACE potential^24^ was refined using the untrapping scheme^59^ in PATHSAMPLE, which attempts to resolve artificial kinetic traps, and was then relaxed with the dispersion correction and at the different pressures.

Considered in isolation, i.e. neglecting the periodicity of the system, the configuration of atoms within the unit cell possesses point group symmetry, and we identify and lump together equivalent permutation-inversion isomers, which are related by operations of the complete nuclear permutation-inversion group under which the Hamiltonian is invariant. Distinguishing these isomers is also possible, but is not necessary here. The lumping operation is performed routinely in the PATHSAMPLE software as part of the optimal alignment procedure.^60,61^ For periodic systems, however, we must also account for an additional degeneracy, which arises from the nonunique choice of unit cell for a given crystal (see, e.g., Figure 3(c) for two equivalent representations of graphite). Rather than Niggli reduction, which may be used to reduce a given representation to a canonical “best” unit cell, equivalent periodic representations of a crystal were identified using a simple, physically motivated algorithm where two cells are considered to correspond to the same structure if their energies, densities, and nearest-neighbor distance profiles match within a chosen threshold, in line with previous work.^17^

## Supplementary Material



## Data Availability

The data that support the findings of this study are available from the corresponding author upon request. OPTIM and PATHSAMPLE are open source code available from https://www-wales.ch.cam.ac.uk/software.html.

## Notes

An illustration of the selection rule for stacking angles in periodic layered systems (PDF)
