# Supplementary material for: An Energy Landscape for Elemental Carbon
Source: J Phys Chem Lett. 2026 Aug 21;17(41):11747–53. doi: 10.1021/acs.jpclett.6c02357 (PMC13679089; doi:10.1021/acs.jpclett.6c02357)
Supplement: Supplementary file 1 [file jz6c02357_si_001.pdf]

Supporting Information for: An Energy Landscape for Elemental Carbon

Vilmos Neuman[1], Soon Woo Park[2], Mark Wilson*[1], and David J. Wales**[2]

[1]Department of Chemistry, Physical and Theoretical Chemistry Laboratory, University of Oxford, South Parks Road, Oxford OX1 3QZ, U.K.
[2]Yusuf Hamied Department of Chemistry, University of Cambridge, Lensfield Road, Cambridge CB2 1EW, U.K.

*Email: mark.wilson@chem.ox.ac.uk
**Email: dw34@cam.ac.uk

A layered structure consisting of atoms arranged in planes of (approximately) fixed interlayer spacing, such as graphite, possesses a selection rule for the allowed angles at which these planes may tilt under periodic boundary conditions. This selection rule may be realised in terms of simple geometry. The associated barrier arises from the requirement that the periodic image of a plane, here viewed as a line in the yz plane, must continue through the cell boundary for the structure to be a continuous layered system. In other words, there must exist a lattice translation $m\mathbf{b} + n\mathbf{c}$ of the unit cell parallel to the plane. Writing the in-plane direction of a plane tilted by θ from the xy plane as the unit vector

$$\hat{\mathbf{u}} = \hat{\mathbf{y}} \cos\theta + \hat{\mathbf{z}} \sin\theta, \tag{1}$$

we hence require

$$m\mathbf{b} + n\mathbf{c} \parallel \hat{\mathbf{u}}, \tag{2}$$

where $m, n \in \mathbb{Z}$, and $\mathbf{b} = b\hat{\mathbf{y}}$ and $\mathbf{c} = c\hat{\mathbf{z}}$ are the cell vectors along the y and z axes as drawn in Fig. 2 of the main article. The parallelism condition is equivalent to

$$(m\mathbf{b} + n\mathbf{c}) \times \hat{\mathbf{u}} = \mathbf{0}. \tag{3}$$

Distributing the vector product, this result reduces to

$$(mb \sin\theta - nc \cos\theta)\, \hat{\mathbf{x}} = \mathbf{0}, \tag{4}$$

so that we are seeking the integer solutions of

$$\tan\theta = \frac{nc}{mb}. \tag{5}$$

The two configurations of Fig. 2 (which possess very similar and for the sake of argument identical unit cells) correspond to the two lowest-index solutions: $(m, n) = (1, 0)$, i.e. $\theta = 0°$ (the horizontal stacking), and $(m, n) = (1, 1)$, i.e. $\theta = \arctan(c/b) \approx 61°$ (the diagonal stacking). Actually, only these two solutions can accommodate the physical interlayer spacing of graphite. Therefore, if the system was to continuously rotate from one minimum to the other, along the pathway, it would break the in-plane bonding by passing through configurations which violate the selection rule of Eq. 5 and hence there is no reason to expect a simple low energy path that involves such rotation. In Fig. S1 below we show the effect of this selection rule pictorially. We also show the calculated lowest energy (two step) pathway interconverting the two graphitic structures, which instead of rotation proceeds via a complex mechanism with an intervening minimum (labelled as Minimum 2). Note that this path is specific to the small cell that we have considered, and more steps would probably be needed for a larger supercell.

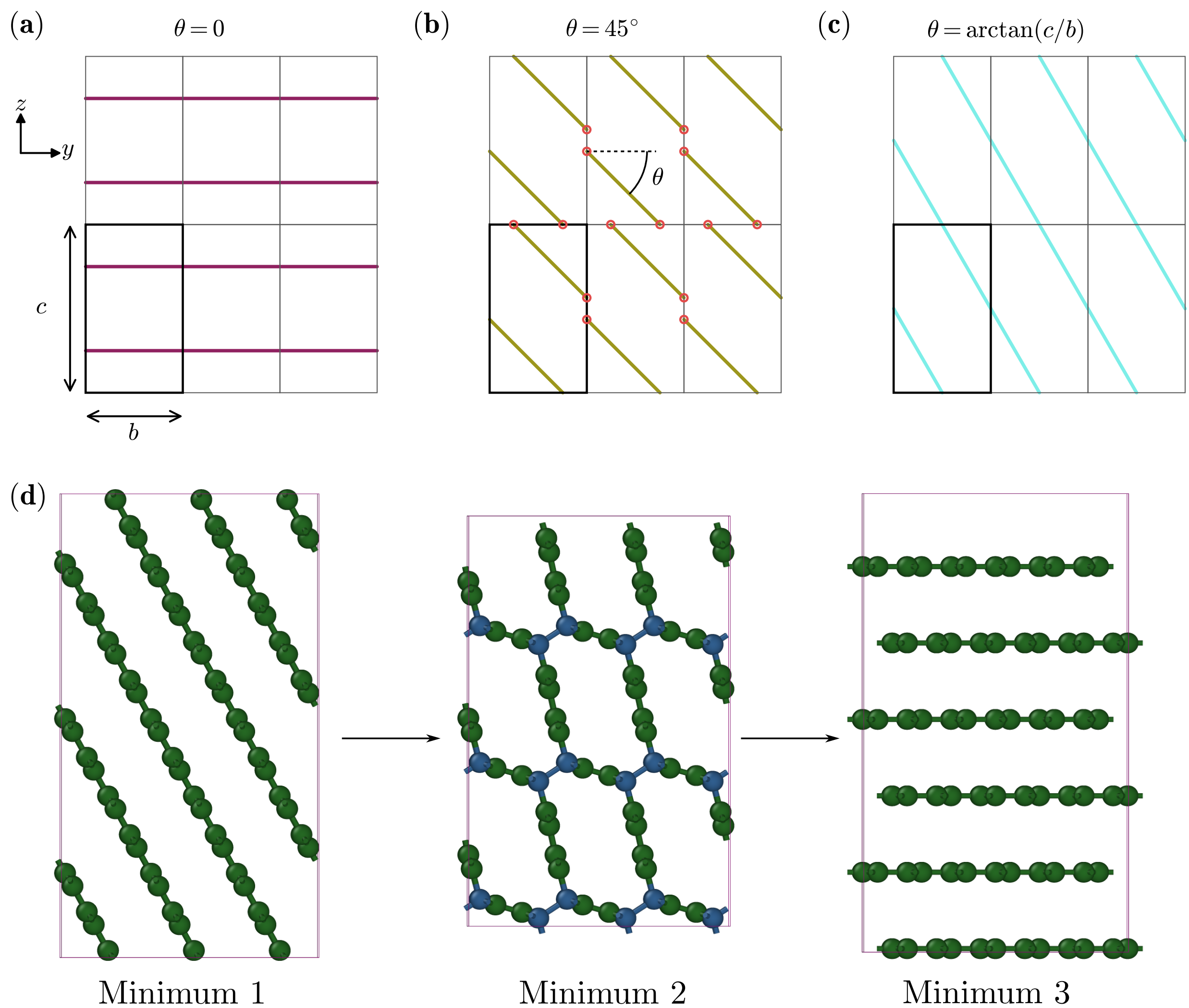

Figure S1: Illustration of the selection rule for stacking angles in periodic layered systems. Panels **a**, **b** and **c** show graphite tilted at zero, 45 degrees and the first non-zero allowed angle, $\theta = \arctan(c/b)$ by Eq. 5 respectively. Panel **d** shows a ball-and-stick model of the lowest energy pathway in the database that interconverts diagonally and horizontally stacked graphite. $3 \times 3 \times 3$ supercells are shown for clarity.
